# Multi-environment QTL studies suggest a role for cysteine-rich protein kinase genes in quantitative resistance to blackleg disease in *Brassica napus*

**DOI:** 10.1186/s12870-016-0877-2

**Published:** 2016-08-24

**Authors:** Nicholas J. Larkan, Harsh Raman, Derek J. Lydiate, Stephen J. Robinson, Fengqun Yu, Denise M. Barbulescu, Rosy Raman, David J. Luckett, Wayne Burton, Neil Wratten, Philip A. Salisbury, S. Roger Rimmer, M. Hossein Borhan

**Affiliations:** 1 Saskatoon Research Centre, Agriculture and Agri-Food Canada, Saskatoon, SK S7N 0X2 Canada; 2 Armatus Genetics Inc, Saskatoon, SK S7W 0C9 Canada; 3 Graham Centre for Agricultural Innovation (an alliance between Charles Sturt University and NSW Department of Primary Industries), Wagga Wagga Agricultural Institute, Wagga Wagga, NSW 2650 Australia; 4 Department of Economic Development, Jobs, Transport and Resources, Grains Innovation Park, Horsham, VIC 3400 Australia; 5 Seednet Australia, Golf Course Road, Horsham, VIC 3402 Australia; 6 Department of Economic Development, Jobs, Transport and Resources, Centre for AgriBioscience, La Trobe University, Bundoora, VIC 3083 Australia; 7 Faculty of Veterinary and Agricultural Sciences, University of Melbourne, Melbourne, VIC 3010 Australia

**Keywords:** *Brassica napus*, *Leptosphaeria maculans*, Blackleg, Quantitative resistance, Chitin, CRK

## Abstract

**Background:**

Resistance to the blackleg disease of *Brassica napus* (canola/oilseed rape), caused by the hemibiotrophic fungal pathogen *Leptosphaeria maculans*, is determined by both race-specific resistance (*R*) genes and quantitative resistance loci (QTL), or adult-plant resistance (APR). While the introgression of *R* genes into breeding material is relatively simple, QTL are often detected sporadically, making them harder to capture in breeding programs. For the effective deployment of APR in crop varieties, resistance QTL need to have a reliable influence on phenotype in multiple environments and be well defined genetically to enable marker-assisted selection (MAS).

**Results:**

Doubled-haploid populations produced from the susceptible *B. napus* variety Topas and APR varieties AG-Castle and AV-Sapphire were analysed for resistance to blackleg in two locations over 3 and 4 years, respectively. Three stable QTL were detected in each population, with two loci appearing to be common to both APR varieties. Physical delineation of three QTL regions was sufficient to identify candidate defense-related genes, including a cluster of cysteine-rich receptor-like kinases contained within a 49 gene QTL interval on chromosome A01. Individual *L. maculans* isolates were used to define the physical intervals for the race-specific *R* genes *Rlm3* and *Rlm4* and to identify QTL common to both field studies and the cotyledon resistance response.

**Conclusion:**

Through multi-environment QTL analysis we have identified and delineated four significant and stable QTL suitable for MAS of quantitative blackleg resistance in *B. napus*, and identified candidate genes which potentially play a role in quantitative defense responses to *L. maculans*.

**Electronic supplementary material:**

The online version of this article (doi:10.1186/s12870-016-0877-2) contains supplementary material, which is available to authorized users.

## Background

Prevention of catastrophic crop loss to plant pathogens is most often achieved through the incorporation of resistance genetics into commercial cultivars. Host responses to plant pathogens are broadly divided into two categories; basal defense responses induced by generic pathogen signals or elicitors called “pathogen-associated molecular patterns” (PAMPs), resulting in mild defense responses collectively known as ‘PAMP triggered immunity’ (PTI) and *R* gene mediated ‘effector triggered immunity’ (ETI) in which race-specific pathogen avirulence (Avr) proteins trigger robust defense mechanisms including hypersensitive response (HR) leading to host cell death at the site of infection [1]. When studying foliar plant pathogens, the HR response of race-specific *R* genes often provides a visual phenotype, indicating an incompatible interaction and allowing for the determination of pathogen virulence. This distinction is used to separate specific *R* gene interactions from quantitative resistance which can provide effective ‘adult plant resistance’ (APR) within a crop variety through the cumulative action of multiple resistance loci. APR is usually measured at the end of the growing season in field trials.

APR is particularly important for combating diseases of *Brassica napus* L. (canola/oilseed rape) in which *R* gene mediated resistance is lacking, such as Sclerotinia Stem Rot (*Sclerotinia sclerotiorum*) [2–4] and Verticillium Wilt (*Verticillium longisporum*) [5–7] or for diseases where pathogen populations often display a rapid adaptation towards *R* gene mediated resistance, such as in the case of blackleg disease, caused by the hemibiotrophic fungal pathogen *Leptosphaeria maculans* [8, 9].

Avoidance of *R* gene mediated resistance by *L. maculans* can occur both rapidly and in a geographically localised fashion when a pathogen population is under heavy selection pressure. A rapid decline in the efficiency of the blackleg *R* gene *Rlm1* in controlling the disease in Europe highlighted the evolutionary potential of the pathogen [10]. A high frequency of mutation and deletion of the *L. maculan*s avirulence gene *AvrLm4*-*7* was reported to occur within a small plot area sown continually to *B. napus* harbouring *Rlm7*, while virulent pathotypes remained undetectable in samples from the surrounding local pathogen population [11]. High rates of infection were observed in some areas of Australia in canola varieties carrying the *R* gene *LepR3* only three years after first commercial release of the material [12], though this rapid loss of effective resistance may have been aided by pre-exposure to *Rlm1* varieties, as avirulence towards *LepR3* and *Rlm1* is conferred by the same *L. maculans* avirulence gene; *AvrLm1* [13].

*B. napus* cultivars containing only APR usually show no difference in the development of leaf lesions when compared with susceptible cultivars, yet they restrict the development of internal stem infection by the pathogen, resulting in lower levels of crown canker formation [14]. This is in contrast to *R* gene mediated resistance which leads to arrest of *L. maculans* growth at the site of infection on cotyledons and leaves. When major *R* gene mediated resistance is avoided by virulent strains within the mixed pathogen population, APR reduces the selection and proliferation of virulent pathotypes in crop residues and the potential for catastrophic crop loss in following seasons [15–17].

While *R* gene mediated resistance can often be detected efficiently and rapidly by observing hypersensitive response after inoculation of *B. napus* cotyledons with well-characterised *L. maculans* isolates, assessment of APR is much more difficult. Resistance needs to be measured either through field-based studies, or under controlled conditions through infection with single spore-derived *L. maculans* isolates and assessment of stem infection in plants grown for several months [18, 19]. Assessment of APR in field-based studies can be difficult considering the complexity of plant-pathogen-environment interactions. Populations of *L. maculans* in most disease nurseries are genetically heterogeneous mixtures arising from sexual recombination and variation of pathotypes should be expected both within a trial site and between trial years. Also, variation of host response due to heterozygosity of *B. napus* lines may be confused for polygenic control of resistance [20]. There has also been a widely-held view that blackleg APR is race non-specific [17], based largely on experience of the French variety Jet Neuf, which provided durable resistance to blackleg disease over many years in Europe and was also utilised in early efforts to improve blackleg resistance in Australian germplasm [21, 22]. However, more recent studies utilising single *L. maculans* isolates have questioned the “race non-specific” nature of blackleg APR [19, 23].

Maintenance of strong APR in canola varieties can most efficiently be achieved through marker-assisted breeding based on the molecular characterisation of quantitative trait loci (QTL) associated with resistance [17]. The French variety Darmor, derived from Jet Neuf, is the most extensively studied *B. napus* variety harbouring quantitative resistance to *L. maculans*. A doubled-haploid (DH) population produced from a cross between Darmor-*bzh* and the susceptible Korean cultivar Yudal (DY) was utilised to map 10 QTL contributing to blackleg resistance, with four of the QTL detected stably across two years of field testing [24]. The resistance was further analysed in Darmor x Samouraï (DS) DH and F_2_ populations, revealing four QTL that were common to both the DY and DS populations [25]. Near-isogenic lines (NILs) were also produced for four Darmor QTL; *LmA2*, *LmA9*, *LmC2* and *LmC4*, though only *LmA2* was fully validated as having a significant effect on reducing disease severity [26]. Blackleg APR has also been assessed in several Australian varieties, revealing multiple QTL that are potentially common to several Australian and French cultivars [9, 19, 27].

Little is known about the molecular basis of APR to *L. maculans* infection in *Brassica* species. While two race-specific genes responsible for ETI-mediated blackleg resistance, *LepR3* and *Rlm2*, have been cloned from *B. napus* and shown to encode extracellular leucine-rich repeat (eLRR) receptor-like proteins recognising the *L. maculans* effectors AvrLm1 and AvrLm2, respectively [13, 28, 29], no genes underpinning blackleg resistance QTL have been identified. Infection of *B. napus* by *L. maculans* results in attempted physical restriction of the pathogen by the host, via callose deposition, while an increased lignification response has also been reported for APR varieties [30, 31]. *L. maculans* infection triggers induction of the salicylic acid (SA) signalling pathway [31, 32] which plays a critical role in plant defense [33]. SA signalling can be triggered in *B. napus* by purified *L. maculans* cell wall components [34] and is greatly induced during ETI, along with the ethylene signalling pathway and H_2_O_2_ accumulation [31, 32, 35]. However, these studies have all focused on early infection events in the cotyledons of *B. napus* seedlings; nothing is known about which defense mechanisms may be active against the invading hyphae as they grow asymptomatically through the petiole [18] and stem [36].

In this study we identified several stable blackleg resistance QTL, with resistance alleles derived from AG-Castle and AV-Sapphire, two blackleg-resistant Australian *B. napus* varieties released in 2002 and 2003, respectively. We used Topas/AG-Castle (TC) and Topas/AV-Sapphire (TS) DH populations to assess the APR of the varieties over multiple years at two locations, performed both single- and multi-environment QTL mapping and defined the physical locations of the QTL relative to the recently released *B. napus* Darmor-*bzh* reference genome [37], allowing for the identification of candidate defense-related genes.

## Results

### Population data

**Fig. 1:**
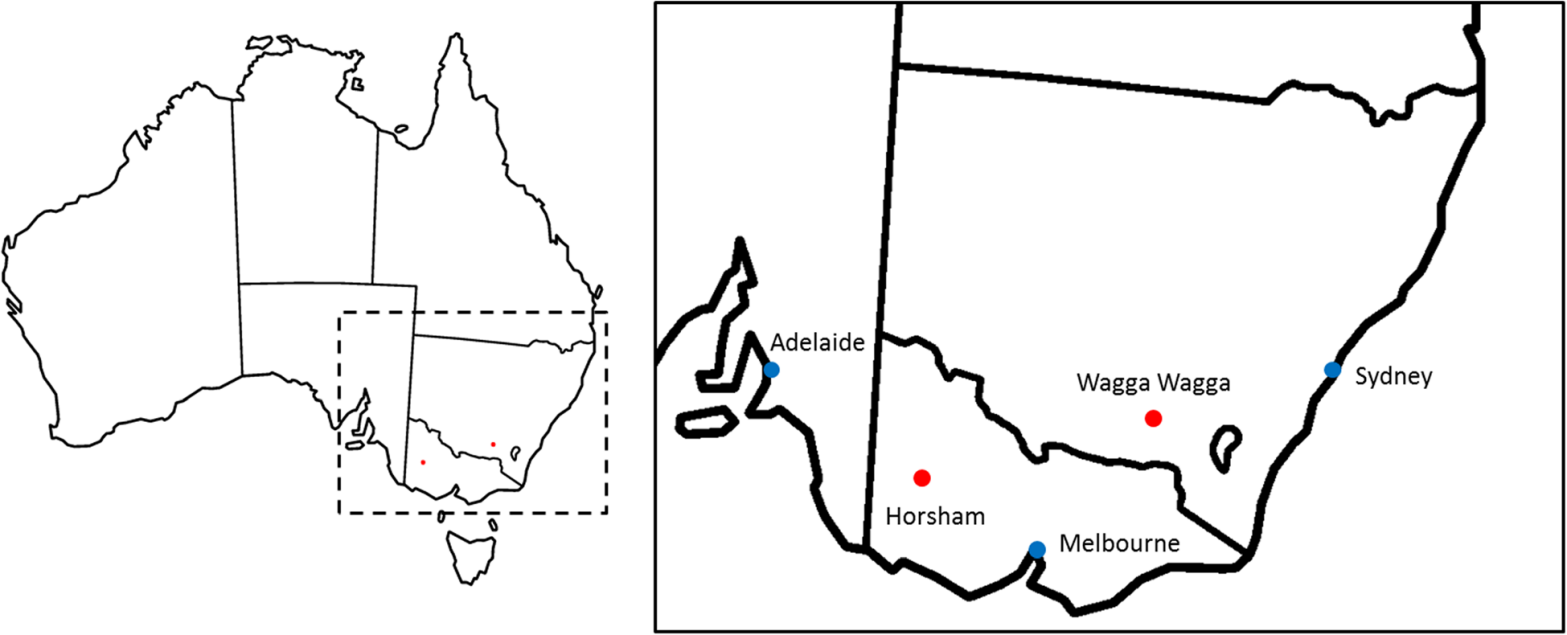
Location of field trial sites in south eastern Australian. *Dashed box* on *left* indicates highlighted region on *right*. *Red dots* show location of trial sites, *blue dots* show major cities. Map modified from original image (https://commons.wikimedia.org)

Field tests were conducted in south eastern Australia in disease nurseries located near Horsham, Victoria and Wagga Wagga, New South Wales (Fig. 1). Mean survival percentages (S) ranged from 25.9 to 43.2 % for the TC population, and 11.2 to 69.2 % for the TS population, with S of individual entries (3 to 4 entries per DH line) ranging from 0 to 100 % in all tests except for TS Horsham 2008, where the maximum S recorded for a single entry was 71.1 %. Mean internal infection percentages (II) ranged from 38.5 to 58.6 % for the TC population and 45 to 87.4 % for the TS population. The minimum II observed was 4 % (TC Wagga Wagga 2010 and TS Wagga Wagga 2009) with a maximum II of 100 % recorded in all tests. For both populations, mean survival was always higher, and mean internal infection was always lower, in tests at the Wagga Wagga site when compared to the Horsham site (Table 1, Additional file 1: Figure S1). Heritability was calculated based on total entries in each environment for each scoring metric; survival (S) and internal infection (II) and was generally high, producing similar ranges for each metric (S: 0.75–0.9, II: 0.73–0.89) (Table 1).

**Table 1:** Survival, internal infection and heritability of DH populations in two environments

Population	Location	Year	Blocks	Metric	Range (%)	Mean (%)	Med.(%)	σ^2^_A_	σ^2^_E_	*h* ^2^
Topas/	Horsham	2009	3	S	0–100	25.9	20	1199.6	148.7	0.89
AG-Castle				II	12–100	58.6	56	1616.2	277.4	0.85
	Wagga Wagga	2009	3	S	0–100	43.2	43	590.5	197	0.75
				II	12–100	56.2	56	729.5	193	0.79
	Wagga Wagga	2010	4	S	0–100	38.6	38	1106.4	165.9	0.87
				II	4–100	38.5	36	741.8	120.7	0.86
Topas/	Horsham	2008	3	S	0–71.1	11.2	6.8	419	44.5	0.90
AV-Sapphire				II	8–100	62.9	64	1937	263	0.88
	Wagga Wagga	2009	3	S	0–100	32	28	1069	223.4	0.83
				II	4–100	45.4	40	941.4	348.7	0.73
	Wagga Wagga	2011	4	S	0–100	69.2	72.8	1325.2	205.1	0.87
				II	16–100	45	44	632.2	123.4	0.84
	Horsham	2012	4	S	0–100	16.5	9	1051.8	155.7	0.87
				II	24.2–100	87.4	94	700.4	85.1	0.89

Data given for each population (Topas/AG-Castle or Topas/AV-Sapphire) in each environment (location x year)

Blocks represent replicates per trial, scoring metrics; *S* survival, *II* internal infection

Range, mean and median (Med.) given for total entries (blocks x DH lines). *σ²*_A_ = variance (additive), *σ²*_E_ = variance (environmental); *h* ^2^ = heritability (*σ²*_A_/*σ²*_A_ + *σ²*_E_)

### Linkage analysis

While the lack of heterozygosity in the populations showed the lines produced to be true doubled haploids, marker distortion was detected in many regions. 40 % of TC marker bins and 20.8 % of TS markers showed mild distortion (failed to conform to a 1:1 ratio; Chi-square test, *p* = 0.05 to 0.001), while 21 % of TC markers and 6 % of TS markers showed severely distorted segregation (*p* < 0.001). The severely affected regions were generally towards the ends of chromosomes; in the TC population they were the upper portions of chromosomes A02, C01, C03 and C09, and the lower portions of A05, C01 and C06. Additionally, the entire A07 chromosome was uniformly distorted, with all markers favoring the Topas parent allele in an approximately 3:1 ratio. In the TS population, upper C03, lower A08 and all of C02 were severely distorted. Two draft maps were initially produced for each population; one containing all markers, and a second in which all severely affected marker loci were removed. Whole-genome QTL analysis was performed using both draft map versions. No changes to QTL positions were observed between draft maps, nor were significant differences in QTL LOD and variance scores, with the exception of the *Rlm3* locus, positioned on A07 on the ‘distorted’ TC map and absent from the ‘non-distorted’ map. To accommodate mapping of the A07 *Rlm3* resistance locus and its associated QTL in the final analysis, TC A07 was resolved independently and added to final TC map. All other distorted markers were removed prior to final map construction. The final TC map consisted of 307 marker bins (collections of co-segregating markers) spanning 2182.3 cM in 21 linkage groups (LGs) representing all 19 *B. napus* chromosomes. Linkage mapping for the TS population produced 23 LGs (199 bins, 1714.97 cM), which were assigned to 18 of the 19 chromosomes, with no representation for chromosome C02.

### Single-environment QTL

**Fig. 2:**
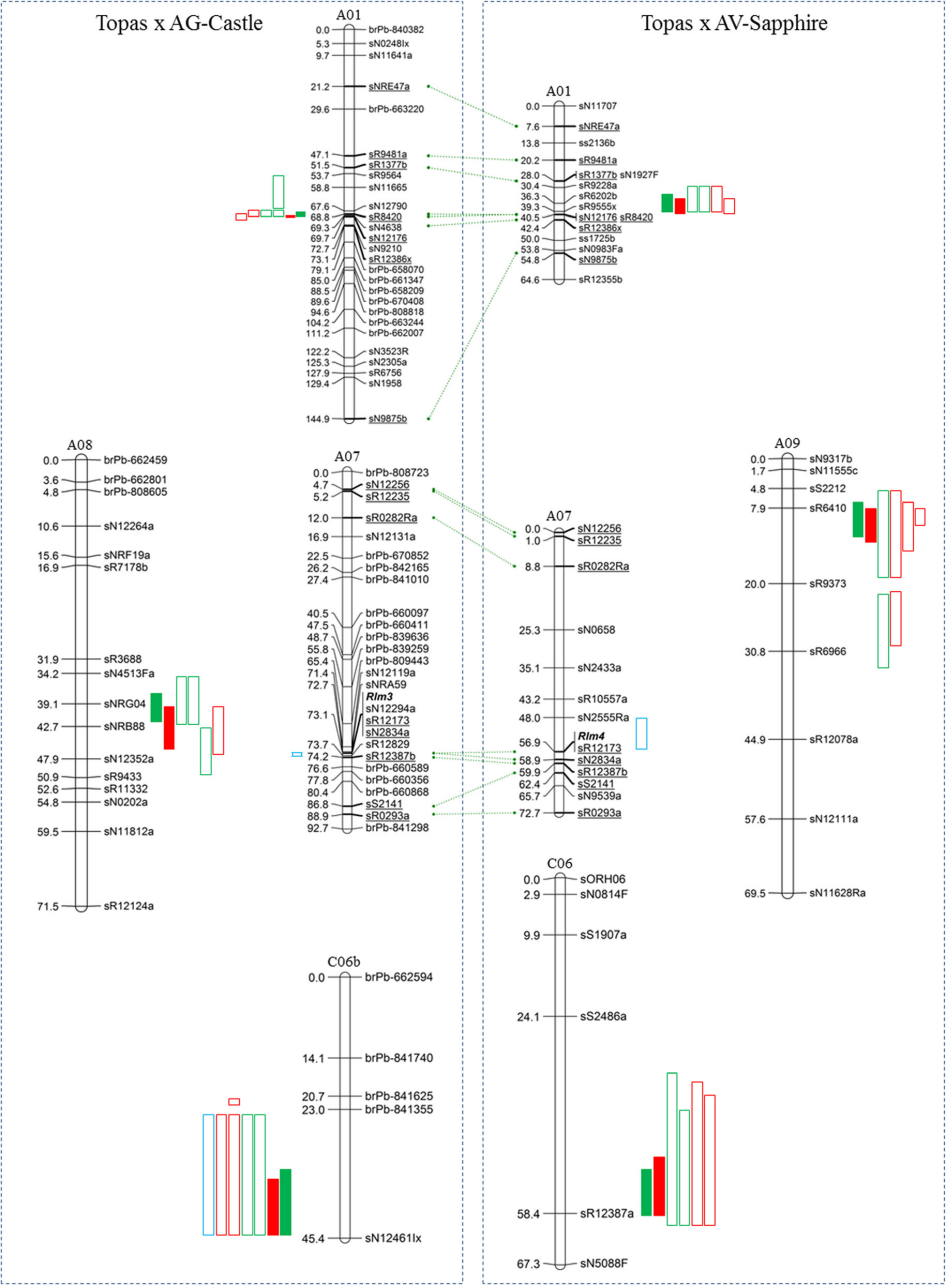
Significant QTL clusters for TC and TS Populations. Linkage maps shown only for *B. napus* chromosomes harbouring significant QTL (TC: A01, A07, A08 & C06; TS: A01, A07, A09, C06). QTL for survival (*green*), internal infection (*red*) and cotyledon (*blue*) metrics. Single-environment QTL shown as *open boxes*, multi-environment QTL as *solid boxes*. *Green dotted lines* indicate common markers. Positions of blackleg *R* genes *Rlm3* and *Rlm4* shown in *bold*

Permutation tests performed for each scoring metric, survival (S) and internal infection (II) percentage, in each environment determined significant LOD thresholds between 2.76 and 3.07 for the TC population and 2.66–2.95 for the TS population. Analysis of the single-environment data produced multiple QTL exceeding their respective LOD thresholds (LOD 3.37–41.47), which were localised to seven chromosomes for the TC population (A01, A08, C03, C04, C05, C06 and C07) and five chromosomes of the TS population (A01, A03, A09, C01 and C06), and accounting for between 2.48 and 31.77 % of the phenotypic variance (Additional file 2: Table S1). To identify chromosomal regions harbouring ‘stable’ and significant QTL regions, QTL identified from individual environments were only considered significant if they exceeded both the LOD threshold for each analysis, based on permutation test (1000 permutations, 0.05 error) and accounted for >5 % of the variance. After applying these criteria three significant QTL, each with favorable alleles derived from the respective resistance donor parent (AG-Castle or AV-Sapphire), were identified in each population. These regions consisted of clustered QTL located on chromosomes A01, A08 and C06 of AG-Castle, and A01, A09 and C06 of AV-Sapphire, with the A01 and C06 QTL regions appearing to be common to both resistance donor parent lines (Table 2, Fig. 2). For the TC larger population (242 lines), the three clustered QTL regions were represented by QTL detected from all three single-environment analyses (Horsham 2009, Wagga Wagga 2009 & 2010), accounting for 8.32 to 31.77 % (A01), 7.05 to 9.08 % (A08) and 5.17 to 14.08 % (C06) of the phenotypic variance. For the TS population (109 lines), only the A09 QTL region was represented by QTL detected in all four tested environments (Horsham 2008 & 2012, Wagga Wagga 2009 & 2010). The TS A01 QTL region was represented by QTL detected in the two 4-block trials (Wagga Wagga 2011, Horsham 2012) which accounted for 9.00 to 18.47 % of the variance in those years. An additional A01 QTL (11.60 % variance) was also detected from the Wagga Wagga 2009 trial (3-block) though this failed to exceed the LOD threshold for significance. The TS C06 QTL region was only represented by QTL detected in the two Horsham nurseries (2008 & 2012), though a large portion of the variance was attributed to this region in those tests (13.63 to 22.18 %).

**Table 2:** Clustered single and multi-environment QTL detected in TC and TS populations

Trait^a^	Chrom.	QTL Int. (cM)	Support interval	Peak (cM)	Peak interval	LOD	σ^2^ (%)	Add	σ^2^_A_	σ^2^_E_	*h* ^2^
A) Topas/AG-Castle											
W09 S	A01	54–67.5	sR9564 - sN12790	59	sN11665 - sN12790	5.20	8.32	4.61			
H09 S	A01	68–69	sN12790 - sN4638	69	sR8420 - sN4638	17.20	15.84	7.80			
W10 S	A01	68–69	sN12790 - sN4638	69	sR8420 - sN4638	24.25	29.90	9.31			
W10 II	A01	68–69	sN12790 - sN4638	69	sR8420 - sN4638	9.08	11.52	4.90			
**MET II**	**A01**	**69**	**sR8420 - sN4638**	**69**	**sR8420 - sN4638**	**34.85**	**20.55**	**6.53**	**12.06**	**8.49**	**0.59**
**MET S**	**A01**	**68.5–69**	**sN12790 - sN4638**	**69**	**sR8420**-**sN4638**	**41.47**	**15.84**	**5.79**	**10.80**	**5.04**	**0.68**
H09 II	A01	69–69.5	sR8420 - sN12176	69.5	sN4638 - sN12176	25.71	31.77	14.01			
CotQTL	A07	73.5–74	sNRA59 - sR12387b	73.5	*Rlm3* - sR12829	72.37	72.18	23.29			
H09 S	A08	34.5–42.5	sN4513Fa - sNRB88	39	sN4513Fa - sNRG04	8.96	7.63	5.49			
W09 S	A08	34.5–42.5	sN4513Fa - sNRB88	39	sN4513Fa - sNRG04	4.45	7.05	4.32			
**MET S**	**A08**	**37.5–42**	**sN4513Fa - sNRB88**	**39**	**sN4513Fa - sNRG04**	**13.59**	**5.13**	**3.51**	**3.85**	**1.28**	**0.75**
H09 II	A08	39.5–47.5	sNRG04 - sN12352a	41	sNRG04 - sNRB88	7.42	7.85	7.08			
**MET II**	**A08**	**39.5–46.5**	sNRG04 - sN12352a	**41.5**	**sNRG04 - sNRB88**	**10.29**	**5.26**	**3.39**	**3.16**	**2.10**	**0.60**
W10 S	A08	43–50.5	sNRB88 - sR9433	46	sNRB88 - sN12352a	8.34	9.08	5.19			
W09 II	C06b	21–22	brPb - 841625 - brPb - 841355	21.5	brPb - 841625 - brPb - 841355	3.04	5.17	3.50			
H09 S	C06b	23.5–45	brPb - 841355 - sN12461Ix	40	brPb - 841355 - sN12461Ix	14.59	14.08	7.42			
CotQTL	C06b	23.5–45	brPb - 841355 - sN12461Ix	40	brPb - 841355 - sN12461Ix	4.71	9.26	7.25			
**MET S**	**C06b**	**36.5–45**	**brPb - 841355 - sN12461Ix**	**42.5**	**brPb - 841355 - sN12461Ix**	**25.99**	**9.22**	**5.06**	**8.12**	**1.10**	**0.88**
W10 II	C06b	23.5–45	brPb - 841355 - sN12461Ix	44.5	brPb - 841355 - sN12461Ix	5.88	7.35	3.94			
H09 II	C06b	23.5–45	brPb - 841355 - sN12461Ix	45	brPb - 841355 - sN12461Ix	10.44	11.07	8.34			
W10 S	C06b	23.5–45	brPb - 841355 - sN12461Ix	45	brPb - 841355 - sN12461Ix	10.00	10.83	5.65			
**MET II**	**C06b**	**38–45**	**brPb - 841355 - sN12461Ix**	**45**	**brPb - 841355 - sN12461Ix**	**16.44**	**7.83**	**4.30**	**5.14**	**2.70**	**0.66**
B) Topas/AV-Sapphire											
H12 S	A01	30.5–39	sR9228a - sR9555x	36.5	sR6202b - sR9555x	4.3901	10.10	5.33			
**MET S**	**A01**	**33.5–39**	**sR9228a - sR9555x**	**36.5**	**sR6202b - sR9555x**	**13.1741**	**10.07**	**5.04**	**8.71**	**1.36**	**0.86**
W11 II	A01	30.5–39	sR9228a - sR9555x	37.5	sR6202b - sR9555x	3.4382	11.07	4.39			
W11 S	A01	30.5–39	sR9228a - sR9555x	39	sR6202b - sR9555x	6.352	18.47	8.21			
H12 II	A01	36.5–40	sR6202b - sN12176	39.5	sR9555x - sN12176	3.3711	9.00	4.08			
**MET II**	**A01**	**36.5–40**	**sR6202b - sN12176**	**40**	**sR9555x - sN12176**	**7.8313**	**5.02**	**4.20**	**4.74**	**0.28**	**0.94**
CotQTL	A07	48–56.5	sN2555Ra - *Rlm4*	56	sN2555Ra - *Rlm4*	30.80	48.92	19.16			
W11 II	A09	5–19.5	sS2212 - sR9373	8	sR6410 - sR9373	5.35	16.38	5.41			
H12 II	A09	6.5–14.5	sS2212 - sR9373	8	sR6410 - sR9373	3.05	8.12	3.92			
W11 S	A09	5–19.5	sS2212 - sR9373	8	sR6410 - sR9373	5.34	15.00	7.48			
**MET S**	**A09**	**6.5–12.5**	**sS2212 - sR9373**	**8**	**sR6410 - sR9373**	**10.41**	**9.18**	**4.67**	**7.33**	**1.86**	**0.80**
W09 II	A09	8–11.5	sR6410 - sR9373	8.5	sR6410 - sR9373	2.89	11.83	6.68			
**MET II**	**A09**	**8–14**	**sR6410 - sR9373**	**9.5**	**sR6410 - sR9373**	**13.97**	**9.66**	**5.88**	**9.10**	**0.56**	**0.94**
H08 II	A09	21–29	sR9373 - sR6966	25	sR9373 - sR6966	3.43	12.31	9.82			
H12 S	A09	21.5–33.5	sR9373 - sR12078a	27.5	sR9373 - sR6966	3.41	9.47	5.19			
H08 II	C06	38–60	sS2486a - sN5088F	52.5	sS2486a - sR12387a	4.79	19.31	12.24			
H12 S	C06	35.5–60	sS2486a - sN5088F	54.5	sS2486a - sR12387a	6.91	22.18	7.88			
H12 II	C06	40.5–60	sS2486a - sN5088F	56.5	sS2486a - sR12387a	4.55	13.63	5.00			
**MET II**	**C06**	**50.5–58.5**	**sS2486a - sN5088F**	**57**	**sS2486a - sR12387a**	**10.84**	**9.67**	**4.97**	**6.69**	**2.98**	**0.69**
H08 S	C06	43–60	sS2486a - sN5088F	58	sS2486a - sR12387a	4.58	15.26	4.73			
**MET S**	**C06**	**53.5–58.5**	**sS2486a - sN5088F**	**58**	**sS2486a - sR12387a**	**14.65**	**9.34**	**5.04**	**8.76**	**0.58**	**0.94**

Significant QTL shown only (Single environment QTL > 5 σ^2^(%), MET QTL > 5 σ^2^(%) and *h* ^2^ > 0.5)

^a^Single environment (plain) and multi-environment (bold) QTL, single-environment trait names given as location (H = Horsham, W = Wagga Wagga), year (08–12 = 2008–2012) and metric (S = survival, II = internal infection), MET = Multi-environment traits (all environments) for S (survival) and MET II (internal infection) metrics, CotQTL = single-isolate cotyledon tests. Chrom. = *B. napus* chromosome; QTL Int. (cM) = QTL interval (in centiMorgans); Support Interval = map interval, defined by flanking markers, which contains QTL (LOD > significance threshold); Peak (cM) = Position of Peak LOD value (in centiMorgans); Peak Interval = map interval containing QTL peak LOD; LOD = peak logarithm of odds; σ^2^(%) = variance (total percentage); Add = additive effect (positive score indicates net genetic contribution from AG-Castle or AV-Sapphire parent); *σ²*_A_ variance (additive) portion (%), *σ²*_E_ variance (environmental) portion (%), *h* ^2^ heritability (*σ²*_A_/*σ²* (%))

### Multi-environment QTL

Multi-Environment Trait (MET) analysis performed using S and II data for all environments produced MET QTL corresponding to each of the seven chromosomes previously identified from the single-environment analysis, except for TC A10 and C07. In addition, multiple low-variance MET QTL were detected for the TS population. Estimates of the additive genetic (A) and environmental (E) components of the multi-environment variance (σ^2^) were used to calculate the narrow-sense heritability (*h*^2^) of each MET QTL. As doubled-haploid populations were used for the study there was no heterozygosity and thus no dominance component for the variance. *h*^2^ values between 0.59 and 0.88 were determined for the TC QTL, while slightly higher *h*^2^ values (0.69–0.94) were determined for QTL in the smaller TS population (Additional file 2: Table S1). Multi-Environment Traits (MET) were considered significant, and the associated chromosomal regions harbouring MET QTL were considered to make a stable contribution to resistance, if they accounted for greater than 5 % of the total variance and were attributed a heritability score of 0.5 or higher. After applying these criteria only MET QTL associated with the previously-identified single-environment QTL clusters were retained. The total multi-environment survival (MET S) and internal infection (MET II) variance explained by the major QTL regions was 30.19 and 33.64 %, respectively, for the TC population, and 28.59 and 24.35 %, respectively, for the TS population (Table 2).

### Single-isolate characterisation

Each DH line in the TC and TS populations were characterised for the presence or absence of hypersensitive response via cotyledon infection tests, as differential phenotypic reactions were initially observed to the *L. maculans* isolates WA30 (avirulent on AG-Castle, virulent on AV-Sapphire) and v23.1.3 (virulent on AG-Castle, avirulent on AV-Sapphire). After the phenotypic data was converted to Mendelised resistant (+) and susceptible (−) scores and incorporated into the genetic maps for each population, the cotyledon resistance loci were determined to localise to chromosome A07, co-segregating with the simple sequence repeat (SSR) marker sR12173 in both cases (Fig. 2). As chromosome A07 is known to harbour the race-specific blackleg resistance genes *Rlm1*, *Rlm3*, *Rlm4*, *Rlm7* and *Rlm9* [19, 38–40] further characterisation of the parental lines with differential *L. maculans* isolates varying in their reactions to the A07 *R* genes was performed. Only isolates avirulent towards *Rlm3* or *Rlm4* produced resistant reactions on AG-Castle or AV-Sapphire, respectively (Table 3). Additional evidence for the presence of *Rlm4* in AV-Sapphire was produced using the transgenic isolate 3R11: *AvrLm4-7*, which demonstrated the *AvrLm4-7* gene conveys avirulence towards AV-Sapphire (Table 3, Additional file 3: Figure S2).

**Table 3:**
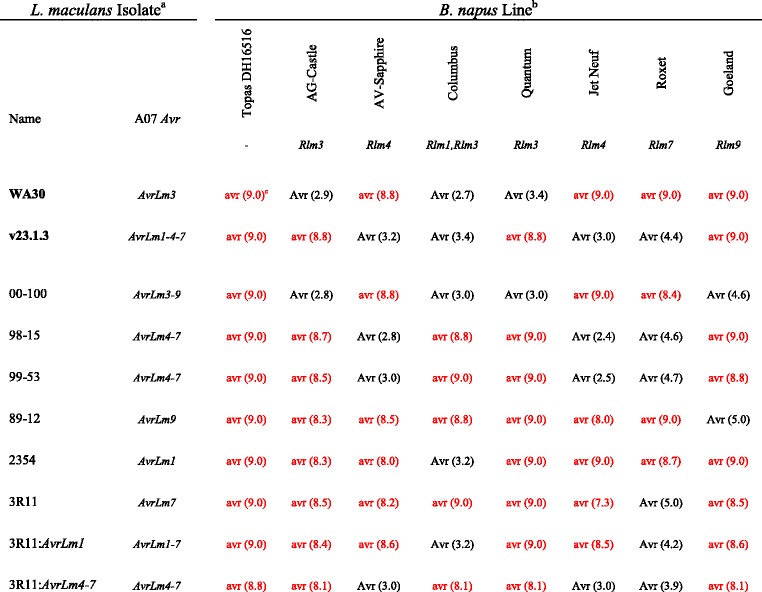
Determination of A07 blackleg *R* genes in AG-Castle and AV-Sapphire

^a^ *L. maculans* isolates used in this study; name followed by avirulence genes corresponding to *Brassica* A07 *R* genes carried by each isolate. 3R11: *AvrLm1* and 3R11: *AvrLm4-7* are transgenic isolates of 3R11 carrying additions of *AvrLm1* and *AvrLm4-7*, respectively. Isolates in bold were used for TC and TS population cotyledon assays

^b^ *B. napus* lines used in the study; name followed by blackleg *R* gene content of line

^c^Interaction of isolate and *B. napus* line; avr = virulence, Avr = avirulence, followed by mean cotyledon rating (in brackets) on 0–9 scale. Entries in red indicate a virulent interaction

The *Rlm3* locus of the TC population co-segregated with a group of three SSR alleles; sR12294a, sR12173 and sR2834a (Fig. 2), which span a region of 80 genes of the *B. napus* Darmor-*bzh* reference genome [37] on chromosome A07 (BnaA07g20270D to BnaA07g21070D). Unfortunately the closest flanking markers to the *Rlm3* cluster were the SSR markers sNRA59 and sR12829, both of which match to portions of the genomic sequence which are not currently incorporated into the *B. napus* chromosome A07 model (matches to ‘chrUn_random’ and ‘chrA07_random’, respectively). The next-closest flanking markers, sR12119a (closest gene = BnaA07g17900D) and sR12387b (BnaA07g26220D) represent a region spanning approximately 4.5 Mb and 832 genes of *B. napus* chromosome A07. Utilising the *B. rapa* genome sequence [41] we were able to define the smaller sNRA59-sR12829 interval as spanning the genes Bra003406 through Bra004064 (658 genes) on *B. rapa* A07, and to infer an equivalent *B. napus* A07 interval of not greater than BnaA07g18610D to BnaA07g24890D (3.5 Mb, 628 genes). This suggested that approximately 30 more genes within the *B. napus* interval were not currently incorporated in the current *B. napus* A07 chromosome build [37].

The mapping of *Rlm4* with the TS population placed the gene between the SSR markers sN2555Ra and sN2834a, a region spanning approximately 3.9 Mb and 704 genes on chromosome A07 (BnaA07g14030D–BnaA07g21070D) which overlapped the *Rlm3* interval defined in the TC population.

During the phenotypic screening of the mapping populations with single isolates, some intermediate phenotypes (scores between 4.1 and 6.9 on the 0–9 scale) were observed. Therefore, to test for QTL affecting the expression of the cotyledon resistance phenotype when challenged with single isolates of *L. maculans*, the phenotypic data was again analysed, this time as continuous data (0 to 9) rather than the discrete “Mendelised” data (‘+’ or ‘−’) analysed previously. As expected, large portions of the variance were associated with each major resistance gene locus (*Rlm3* and *Rlm4*). We also detected a second significant QTL for the TC population which accounted for 9.26 % of the cotyledon phenotypic variance and co-localised with the TC C06 QTL cluster (Table 2, Fig. 2).

### Delineation of QTL loci and identification of candidate genes

The 0.5 cM map interval containing the TC A01 MET S and MET II loci was flanked by the markers sN12790 and sN4638 (Fig. 2), which corresponds to a span of 49 *B. napus* genes (BnaA01g12170D–BnaA01g12660D). The peak MET LOD scores for each metric were flanked by the markers sR8420 and sN4638 which corresponded to a 10 gene interval of the Darmor-*bzh* reference *B. napus* genome (BnaA01g12560D–BnaA01g12660D). The TS A01 QTL locus was flanked by the markers sR9228a and sN12176, representing a span of 296 genes (BnaA01g09950D to BnaA01g12910D), with peak LODs for all QTL contained with the 4.2 cM marker interval sR6202b–sN12176 (BnaA01g10980D to BnaA01g12910D), representing a span of 193 *B. napus* genes which also encapsulates the physical interval defined for the TC A01 QTL locus.

The TC population produced a cluster of single- and multi-environment QTL on chromosome A08, with peak values for all QTL positioned between 39 and 46 cM (Table 2). The multi-environment analysis defined overlapping MET S and MET II QTL contained within a marker interval of 13.7 cM flanked by the markers sN4513Fa and sN12352a. This interval corresponds to a region of 396 genes in the Darmor-*bzh* reference genome (BnaA08g18290D–BnaA08g22250D).

The QTL cluster detected for the TS population on chromosome A09 was positioned within the marker interval sS2212 to sR9373, which corresponds to a physical interval of 1314 genes in the *B. napus* reference genome (BnaA09g22470D–BnaA09g35610D). While the peak LOD for many of the QTL, including both the MET S and MET II QTL, was positioned within the smaller sR6410–sR9373 marker interval, the physical region could not be refined any further using the *B. napus* reference genome, as sR6410 was assigned to the ‘chrA09_random’ molecule which is not incorporated into the main A09 chromosome build. However, by using the *B. rapa* Chiifu A genome reference sequence [41], we identified homology between sR6410 and the *B. rapa* A09 gene Bra006927 and used a neighbouring gene (Bra006925) to determine an approximate *B. napus* physical position for this gene as equivalent to BnaA09g32910D of *B. napus*. This produced a physical delineation for the A09 QTL peak interval of approximately 270 genes (BnaA09g32910D–BnaA09g35610D).

QTL clusters were also detected on chromosome C06 in each population, though poor resolution of these linkage groups hampered precise delineation of the support intervals. The TS C06 QTL support interval was delimited to a 2998 gene span of the *B. napus* genome (BnaC06g05190D–BnaC06g35170D) between the markers sS2486 and sN5088F while in the TC population, a *B. napus* physical interval could not be defined as the upper flanking marker for the TC C06 support interval (DArT marker brPb-841355) provide a match to the unincorporated ‘chrUn_random’ molecule. We were, however, able to define a syntenic physical interval in the *B. oleracea* reference genome [42] of approximately 9 Mb on C06 (1,797,307..10,864,498), which contains 1336 predicted genes.

Delineation of the TC A01, TC A08 and TS A09 QTL loci produced physical intervals sufficiently small to warrant identification of gene candidates that had potential roles in underpinning pathogen resistance QTL. The TC A01 QTL peak LOD interval spanned only ten genes, which straddled a cluster of eight genes (BnaA01g12580D–12590D, BnaA01g12610D–12630D, BnaA01g12650D–12670D) with homology to the Cysteine-rich Receptor-Like Protein Kinase (CRK) genes of *A. thaliana* chromosome 4 (At4g23190, At4g23300 and At4g04570, respectively). CRKs are one of the largest super-families of receptor kinases in Arabidopsis with 44 members [43], several of which have been implicated in plant defense responses [44–49]. The TC A08 and TS A09 peak QTL intervals span 396 and 270 genes within the reference *B. napus* genome, respectively. Within these spans are several potential resistance-related genes including receptor-like proteins, a receptor-like kinase and TIR-NB-LRR homologues.

## Discussion

We describe here the detection and characterisation of highly-stable quantitative resistance loci to the *B. napus* fungal pathogen *L. maculans*. Through multi-environment analysis we were able to define highly-heritable resistance loci effective in some of the harshest testing conditions in the world and to identify putative resistance-related genes that are located within the physically-defined QTL regions.

In performing the QTL tests over multiple environments we were able to produce estimates of heritability (*h*^2^: the degree to which genetics determines phenotype) for each MET QTL (Table 2). Traditionally heritability is calculated as a function of the variance within the entire population (Table 1). While this provides an estimate of the genetic influence on the over-all phenotypic variance, this does not provide information on the environmental variability of individual QTL loci within the population. By calculating *h*^2^ values for each MET QTL we can offer an estimate of how environmental variability will affect the phenotypic variation, particularly when targeting individual QTL loci in breeding programs. We also observed that in the larger TC population (242 DH lines), the *h*^2^ values were consistently higher when MET QTL were determined using the survival metric compared to the internal infection metric, though this did not hold true for the smaller TS population (109 lines). This may be due to the influence of ‘escapes’ in the scoring of the field trials. While significant differences were seen in survival between the ‘susceptible’ Topas and ‘resistant’ parental cultivars AG-Castle and AV-Sapphire, the difference was not always evident with the internal infection metric (Additional file 4: Table S2, Additional file 1: Figure S1). Under Australian field conditions, infection is driven by sexual ascospores and often results in seedling death for susceptible plants [50]. This means very few susceptible plants will remain standing at the end of the growing season when internal infection is assessed. However, the remaining survivors have been enriched for escapes i.e. plants that did not develop the same level of disease due to a delay in, or lack of, exposure to the pathogen, particularly when seedling germination is not consistent. This would result in an under-estimate of internal infection for individual lines, as only a portion of the standing plants are assessed. The same effect would not be as significant in the survival metric where the entire row is counted.

The genomic location of the TC A01 QTL interval matches the previously reported position of a blackleg QTL from the Australian cultivar AG-Spectrum [19]. A recent report detailing QTL mapping in European winter oilseed rape populations [51] placed QTL from Grizzly/Bristol and Darmor/Bristol populations within a region of A01 that spans the TC A01 QTL locus defined in our study (approx. BnaA01g08200D–BnaA01g20140D). QTL on chromosome A01 have also previously been reported for several DH populations derived from Australian varieties, including AV-Sapphire [27]. Favourable alleles from the AG-Castle A08 QTL locus (BnaA08g18290D–BnaA08g22250D) are positioned adjacent to the QlmA8_DB QTLs previously identified in the Darmor/Bristol population, which were positioned between the SSR markers BN53449 and sR3688 (BnaA08g12480D–BnaA08g17050D) on chromosome A08 [51], and to the DY A08 QTL detected in the Darmor-*bzh/*Yudal population [26, 52]. Additionally, the TS A09 QTL locus from AV-Sapphire provides a near-complete overlap with the previously-reported syntenic *A. thaliana* region At3g25805–At3g58680 (equivalent to approximately BnaA09g19610D to BnaA09g37480D of the *B. napus* genome), which was identified as syntenic to the *LmA9* QTL interval from the Darmor-*bzh/*Yudal QTL map [26]. Finally, QTLs from Aviso and Darmor/Bristol populations were also placed within a 369 gene interval (BnaC06g31460D–BnaC06g35150D) on lower C06 [51], which is in agreement with the large C06 QTL regions detected in both the TC and TS populations. The correlation of blackleg QTL in many of the studied *B. napus* varieties suggests the overall pool of APR genetics utilised in canola varieties world-wide may be rather limited.

Pedigree analysis for AG-Castle suggests nearly half of the variety’s genetic contribution (46.9 %) is derived from Japanese material, with European material making up the bulk of the remainder (29.8 %) [53]. A study of *B. napus* germplasm diversity has shown the variety AG-Spectrum is closely related to Rainbow [54], an Australian polygenic resistant variety which is also featured in the pedigree for AG-Castle [53]. The *B. napus* variety Major, a progenitor of the well-characterised French APR variety Darmor (Major - > Primor - > Jet Neuf - > Darmor) was grouped into a different clade than Rainbow and AG-Spectrum [54], suggesting a low over-all genomic relationship between sources of APR for the Australian and European QTL studies. However, the correlation of QTL on A01, A08, A09 and C06 between Australian and French varieties demonstrates a high degree of selection and retention of these QTL after the introduction of French APR germplasm into Australian breeding programs [22], suggesting enduring efficacy of these QTL against Australian *L. maculans* populations. However, a “slow erosion of polygenic resistance” has been observed in Australian breeding programs [53] and suggests that future efforts should be focused on the detection and introgression of novel APR genetics from diverse Brassica germplasm rather than the continued recycling of over-used QTL. Interestingly, the *B. juncea* line BJ168 also makes a small contribution (6.3 %) to the pedigree of AG-Castle and many other Australian varieties, though none of the major *R* genes from *B. juncea* have been introgressed into Australian cultivars [53]. These *R* genes, present in the B genome of *B. juncea*, are potentially valuable sources of resistance for *B. napus* breeding if stably introgressed [55–57]. However, little is known of the quantitative resistance potential of the *B. juncea* A genome, which harbours distinct genomic diversity [58], or if *B. juncea* A genome introgressions have contributed to the pool of blackleg APR utilised in Australian germplasm, and this remains a potentially under-utilised resource for resistance genetics.

Investigation of the genomic region defined by the TC A01 peak QTL interval revealed a cluster of cysteine-rich receptor-like kinase genes (CRKs). CRKs are characterised by one or more extracellular C-X8-C-*X*2-C motifs (DUF26/GNK2) that likely mediate protein-protein interactions [43]. CRK genes have been shown to be induced in plants during pathogen infection [44–49], including the upregulation of several CRKs during infection of *B. napus* cotyledons by *L. maculans* [32]. Active early in the plant defence response to pathogens, CRKs are induced independent of other host response genes such as *EDS1* or *NPR1* [44, 45]. While some members of the CRK superfamily have been demonstrated to enhance resistance against pathogens by promoting hypersensitive response (HR) and salicylic acid (SA) accumulation [44, 45], the use of knock-out mutants and transient gene silencing has suggested that some other CRKs can negatively affect defense responses, acting in a quantitative fashion [47, 48]. CRKs have also been shown to have a role in symbiotic plant-microbe interactions, with the *symCRK* gene of the legume *Medicago truncatula* being required for maintenance of functional nodules and the suppression of host defense during the symbiotic process [59]. Recently it was demonstrated that over-expression of some Arabidopsis CRKs enhanced PTI defense responses, such as oxidative burst, stomatal closure and callose deposition, towards the bacterial pathogen *Pseudomonas syringae* pv. *tomato* DC3000. Additionally, these proteins were shown to associate with the pattern-recognition receptor FLAGELLIN SENSING2 (FLS2) at the plasma membrane [60], an essential component of the bacterial pathogen PAMP response [61]. A second study, profiling a near-complete set of Arabidopsis CRK mutants, showed some CRKs respond to fungal chitin, a common PTI elicitor [62, 63], and play a role in regulating stomatal closure [64]. The roles of CRKs in quantitative plant defense responses suggests the cluster of CRK genes co-localised with the TC A01 QTL interval offer viable candidate genes for blackleg APR. Further study of these genes, including their temporal and spatial regulation in response to *L. maculans* infection, is currently underway. While the other QTL intervals defined in this study were not small enough to warrant extensive candidate gene studies, other CRK homologues were found with the TS A09 (BnaA09g23770D) and TS C06 (BnaC06g31680) intervals.

While no stable QTL were detected in which the resistant allele originated from the Topas “susceptible” parent, it should be noted that in most environments Topas had a higher survival percentage than the commonly-used susceptible line Westar (Additional file 4: Table S2). Under the disease nursery conditions in Australia, the Westar rows showed near-complete seedling death, often leaving no plants at all at the end of the growing season, while the Topas rows still produced some adult plants, though most were dead or highly cankered at the time of rating. It is conceivable that Topas contains some small degree of APR common to both AG-Castle and AV-Sapphire, and perhaps many other *B. napus* cultivars, which would not be detectable as QTL in these populations. While some Topas-derived QTL were detected during the study, these were always specific to a single testing environment, usually accounted for less than 5 % of the variance and were not associated with any significant MET QTL (Additional file 2: Table S1). Under the extremely high disease pressure of the Horsham 2012 trial, both Topas and Westar had 0 % survival (Additional file 4: Table S2, Additional file 1: Figure S1), so any minor APR that may have been expressed by Topas was rendered completely ineffective under those conditions.

Our single-isolate characterisation of the TC and TS populations also delimited the physical positions of the major *R* genes *Rlm3* and *Rlm4* to intervals of 3.5 Mb and 3.9 Mb, respectively, on chromosome A07. While these genes have been known to cluster genetically, along with *Rlm7* and *Rlm9*, on A07 for over a decade [38], this is the first report to define a physical location for this cluster in the *B. napus* genome. Previously, Raman et al. (2012c) positioned the *Rlm4* gene (from *B. napus* variety Skipton), assessed at both the cotyledon and adult plant stages, to a 24.8 cM interval of A07 using SNP markers. While the physical interval relative to *B. napus* was not defined in this study, several SNPs corresponding to approximately 15.9–16.9 Mb (~BnaA07g20250D–BnaA07g21900D) on *B. napus* A07 were associated with the *Rlm4* locus [65]. This location would be in agreement with the *Rlm4* physical interval defined for the TS population in this study (BnaA07g14030D–BnaA07g21070D) and the co-segregation of the locus with the SSR marker sR12173 (BnaA07g20910D).

Neither *Rlm3* nor *Rlm4* were found to be associated with QTL detected in the field studies, suggesting a high degree of virulence towards both *R* genes in the populations of *L. maculans* infesting the disease nurseries. This result was not unexpected, as high rates of virulence towards *Rlm3* [66] and *Rlm4* [23] have been previously reported for Australian *L. maculans* isolates. Analysing the cotyledon reactions to single *L. maculans* isolates avirulent towards the respective *R* genes produced high LOD, high variance ‘CotQTL’ loci associated with each gene, as expected. An additional cotyledon-responsive interaction with the isolate WA30 was also demonstrated in the TC population which accounted for 9.26 % of the cotyledon phenotypic variation and localised to the C06 QTL interval (Fig. 2, Table 2). No additional significant cotyledon-responsive QTL were detected in the TS population, which also contains a C06 QTL locus, using isolate v23.1.3. Previous studies have observed a significant difference in *L. maculans* cotyledon lesion development between APR and non-APR varieties [18]. Cotyledon-expressed QTL similar to the one detected in our study have also previously been defined in the SASDH (Skipton/ AG-Spectrum) population and localised on chromosomes A01 and A10, though only the A01 QTL was associated with field resistance in the adult plant. These cotyledon QTL interactions also appeared to be race-specific, having been detected with the *L. maculans* isolate 06MGPP041 but not isolate 04MGPS021 [19]. Blackleg resistance at seedling and adult stages of the host have often been viewed as separate mechanisms; the former governed by race-specific *R* gene interaction in cotyledons and leaves, and the latter by non-specific QTL active in the stem. The detection of common QTL loci in both seedling (cotyledon) and adult plant (field) tests, and the differential interaction of individual *L. maculans* isolates to those QTL, suggests that such distinctions are not always valid. The associated resistance response is likely triggered early in the interaction between host and pathogen, activated long before entry of the pathogen into the stem tissues of the plant. The action of these QTL may be to impair the initial infection process, rather than to actively resist invasion of adult stem tissues, leading to delayed development of stem canker symptoms in the adult plant. If the quantitative blackleg resistance found in *B. napus* is truly “non-race specific” then the entirety of the environmental variance observed in field trials can be attributed to differences in the physical environmental factors (rainfall, temperature etc.) affecting each trial. However, if *L. maculans* isolates react differentially to QTL, then a large component of the environmental variance could be explained by variation in the pathogen populations used in the trials, both between sites and years. Alternatively, as it has been previously shown that *B. napus* blackleg *R* genes require other partner proteins in order to successful trigger the resistance response to *L. maculans* [29, 67], minor QTL detected during cotyledon tests may represent either allelic variation or altered transcriptional patterns for *R* gene interacting proteins. Further dissection of the temporal and spatial effects of quantitative resistance responses during *L. maculans* infection, as well as the effectiveness of these responses against a number of diverse isolates, will aid in bettering our overall understanding of the blackleg resistance response and how best to utilise the available genetics in crop protection.

## Conclusions

As pathogen populations become enriched for virulence against commonly-deployed *R* genes, alternative resistance genetics need to be incorporated into crop breeding programs in order to maintain global agricultural production. The identification and mapping of QTL is required for the efficient introgression of stable resistance genetics in modern MAS breeding programs, enabling the effective utilisation of quantitative genetics in plant protection. In this study we have delineated four stable blackleg resistance QTL through multi-environment QTL analysis. We have identified a cluster of cysteine-rich receptor-like kinase genes associated with a significant field-derived QTL, suggesting a role for these genes in quantitative defense responses to *L. maculans*. The genomic delineation of effective blackleg resistance QTL described in this work should benefit resistance breeding efforts, and further the investigation into the identity of genes controlling APR in *Brassica* germplasm.

## Methods

### Plant populations

F_1_ seedlings, produced by crossing the Swedish spring-type, blackleg-susceptible *B. napus* variety Topas with the Australian blackleg-resistant varieties AG-Castle and AV-Sapphire, were used to produce DH populations via microspore culture by the method of Ferrie et al. [68] using modified Lichter medium (NLN) with 13 % sucrose. The Topas/AG-Castle DH population (TC) utilised for this study consisted of a total of 242 DH lines from two sub-populations of 139 and 103 DH lines, with each sub-population produced from a single F_1_ plant. The Topas/AV-Sapphire DH population (TS) contained a total of 109 lines, also consisting of two sub-populations of 66 and 43 DH lines. Seed of parental and DH lines used in the QTL trials was produced in tented field plots in Saskatoon, Canada during the 2007 (TS) and 2008 (TC) summer growing seasons, packaged into replicates of 100 seeds per line and sent to Australia for testing field resistance to blackleg. The Topas *B. napus* line was sourced from the AAFC Saskatoon collection. AG-Castle and AV-Sapphire lines were provided by DEDJTR Victoria (formerly Department of Primary Industries).

### Field characterisation of DH populations

The parental lines and their respective DH populations, along with several *B. napus* variety checks (Additional file 4: Table S2) were grown May through November in blackleg disease nurseries situated at Wagga Wagga (New South Wales) and two sites (Wonwondah and Green Lake) south of Horsham (Victoria) between 2008 and 2012 (Fig. 1). Rainfall differed by an average of 103.2 mm/year over the three trial years at each site, with the Wagga Wagga site receiving an average 38 % more precipitation (Additional file 5: Table S3). Each trial was planted in a randomised block design with 3 to 4 replicates per line. The TC population was tested in three separate environments; both Wagga Wagga and Horsham (Wonwondah site) in 2009 (3 replicates per trial) and once more at Wagga Wagga in 2010 (4 replicates). The TS population was tested in four environments; Horsham (Wonwondah site), 2008 and Wagga Wagga, 2009 as 3-replicate trials and Wagga Wagga, 2011 and Horsham (Green Lake site), 2012 as 4-replicate trials. Emergence counts were made 4–5 weeks after sowing. For trials at the Horsham sites, local diseased canola stubble, collected in the previous year, was spread as the source of *L. maculans* inoculum, while at Wagga Wagga the trials were sown into *in situ* canola stubble from the preceding season as previously described [19]. At the end of the growing season trials were assessed for blackleg disease using both survival percent (S) and internal infection (II) metrics. S was calculated for each row as (Standing plants at end of season/Emergence count)*100, II determined by examining 5–20 random plants per row for internal infection after cutting the stem at the crown and rating fungal discoloration on a 0–5 scale (0 = no infection, 1 = 1–25 % infection, 2 = 26–50 % infection, 3 = 51–75 % infection and 4 = 76–99 % infection and 5 = 100 % infection/missing plant) and median scores were calculated for each entry and metric using Microsoft Excel. Statistical analysis of populations and plotting of distribution histograms were performed using R software [69].

Selected lines representative of the 3 major QTL identified from the TC DH population in this study, both singularly and in combination, were also entered into Saskatchewan disease nurseries at Melfort (2012) and Saskatoon (2013) in attempt to test the efficacy of the QTL resistance against Canadian populations of *L. maculans*. However, neither site produced sufficient disease pressure to assess the resistance, with Westar control entries presenting average internal infections of less than 25 % at the end of the growing season.

### Single-isolate characterisation

Each DH line in the TC and TS populations were characterised for the presence or absence of hypersensitive response during infection by the single spore-derived *L. maculans* isolates WA30 (Rimmer Collection, AAFC Saskatoon) or v23.1.3 (INRA-Bioger collection, France), respectively, via cotyledon infection tests. Four plants for each line were scored at 14 days post-infection on a 0–9 scale as described by Larkan et al. [13]. Further differential pathology testing, using isolates of *L. maculans* varying in their avirulence gene profiles (00–100, 98–15, 99–53, 89–12, 2354-Rimmer Collection, AAFC Saskatoon; 3R11-Howlett Lab, University of Melbourne, Australia, 3R11: *AvrLm1* [13]) was performed with the parental lines and the *R* gene control lines Topas DH16516 (a DH line of Topas with no effective blackleg resistance), Columbus (*Rlm1*, *Rlm3*), Quantum (*Rlm3*), Jet Neuf (*Rlm4*), Roxet (*Rlm7*) and Goeland (*Rlm9*) in order to produce a hypothesis for the likely *R* gene content of each population (Table 3). Inoculum for the tests was produced as described previously [70]. Spores were diluted to 2x10^7^ spores/mL and 10 μL was used to inoculate each of four small wounds on each plant (one wound at the centre of each cotyledon lobe).

The transgenic isolate 3R11: *AvrLm4–7* was produced by Agrobacterium transformation [71]. Briefly, a 2205 bp amplicon was produced from the isolate v23.1.3 (primers: GW-AvrLm47G-Fc; GGGGACAAGTTTGTACAAAAAAGCAGGCTTCCTCTGGTAAGGAAGGTTTACCAATTATACACCT, GW-AvrLm47G-Rb; GGGGACCACTTTGTACAAGAAAGCTGGGTCGGCGGTAGATTTGCTACTAAAAGGTAACTTT) which spanned 1213 bp upstream and 514 bp downstream of the *L maculans AvrLm4–7* CDS [72] and inserted into the Gateway-compatible fungal transformation vector pNL11 prior to transformation of the *L. maculans* isolate 3R11 as previously described [13].

### Map construction and QTL identification

The TC and TS populations were characterised using 225 and 212 simple sequence repeat (SSR) markers, respectively, as described previously [73]. A subset of the TC population had previously been characterised using 279 DArT markers as part of a consensus map of *B. napus* [74], and this data was also incorporated into the TC map to provide additional resolution and to allow orientation to other maps (503 markers total, grouped into 360 bins). Linkage map construction and inclusive composite interval mapping (ICIM) [75] was performed using QTL IciMapping version 3.3 (http://www.isbreeding.net). Prior to final map construction severely distorted markers (Chi-square Test, *p* < 0.001) were excluded. A threshold of LOD 6 was applied for initial grouping of markers. LGs consisting of > 3 markers were anchored to chromosomes based on BLAST-like alignment tool (BLAT) analysis of SSR marker sequences to the published *B. napus* genome [37] before recalculating LGs at LOD 3. Marker ordering was performed using nearest neighbour algorithm and rippling was performed once using the sum of adjacent recombination fractions (SARF) criterion. For QTL identification, the ‘ICIM-ADD’ and ‘ICIM-EPI’ functions of the software were utilised to investigate both additive and digenic epistatic QTL [76, 77] expressed in each environment and for each scoring metric (S and II). Permutation tests (1000 permutations, 95 % confidence level, 1 cM interval) were performed for each scoring metric in each environment to determine significant LOD thresholds. The precision of QTL positions were then improved by reanalysing the data using the determined LOD thresholds and a smaller (0.5 cM) scan interval. The ‘Multi-Environment Trials’ (MET) function of the software was also utilised to determine the consensus positions for the major QTL and to assess the genotype by environment (GxE) interactions. QTL identified from individual environments were considered significant if they exceeded the LOD significance threshold and accounted for > 5 % of the variance. MET QTL were considered significant if they accounted for > 5 % of the variance and had a heritability (*h*^2^ = σ^2^(Additive)/σ^2^(Total)) > 0.5. QTLs positions were defined by both support and peak marker intervals, with support intervals representing the map interval in which the LOD exceeded the calculated LOD threshold (for single-environment QTL), or peak LOD-1 (for multi-environment analyses). The data sets for each population were analysed both as total populations and as their separate component sub-populations. Final map figures were produced using Mapchart 2.2 [78] and Microsoft PowerPoint software. To determine the location of the major QTL relative to the *B. napus* reference Darmor-*bzh* genome [37] the target sequences for the flanking SSR markers were obtained from the AAFC Brassica MAST Database (http://aafc-aac.usask.ca/BrassicaMAST/) and used to search the public *B. napus* genome database (http://www.genoscope.cns.fr) using default BLAT search settings.

## Additional files

Additional file 1: Figure S1. Distribution of Median Survival and Internal Infection Percentages. Distribution of scores for survival (green bars) and internal infection (red bars) given for each population (TC or TS) in each trial. Environment names given as location (H = Horsham, W = Wagga Wagga), year (08–12 = 2008–2012). Green and red lines indicate mean survival and internal infection percentages for parental lines, respectively. (PNG 127 kb) Additional file 2: Table S1. All single- and multi-environment QTL (LOD >2.5). (DOCX 43 kb) Additional file 3: Figure S2. Transgenic complementation of *Rlm4* in AV-Sapphire. Phenotypic interaction of isolates 00–100 (*avrLm4–7*), v23.1.3 (*AvrLm4–7*), 3R11 (*AvrLm7*) and transgenic isolate 3R11: *AvrLm4–7* (*AvrLm4–7*) with *B. napus* lines Topas (no blackleg *R* genes) and AV-Sapphire (*Rlm4*). (PNG 1656 kb) Additional file 4: Table S2. Survival and internal infection of control *B. napus* lines in each environment. (DOCX 16 kb) Additional file 5: Table S3. Rainfall (mm) for trial sites over growing season (May–Nov). (DOCX 15 kb)

## Abbreviations

APR: Adult plant resistance
BLAT: BLAST-like alignment tool
CRK: Cysteine-rich receptor kinase
DH: Doubled haploid
eLRR: Extracellular leucine-rich repeat
ETI: Effector-triggered immunity
HR: Hypersensitive response
ICIM: Inclusive composite interval mapping
II: Internal infection
LG: Linkage group
LOD: Logarithm of the odds
MAS: Marker-assisted selection
MET: Multi-environment trait
NIL: Near-isogenic line
PAMP: Pathogen-associated molecular patterns
PTI: PAMP triggered immunity
QTL: Quantitative trait loci
S: Survival
SA: Salicylic acid
SNP: Single nucleotide polymorphism
SSR: Simple sequence repeat
TC: Topas/AG-castle
TS: Topas/AV-capphire
