# Supplementary figures and images for: Multi-environment QTL studies suggest a role for cysteine-rich protein kinase genes in quantitative resistance to blackleg disease in Brassica napus
Source: BMC Plant Biol. 2016 Aug 24;16(1):183. doi: 10.1186/s12870-016-0877-2 (PMC4995785; doi:10.1186/s12870-016-0877-2)

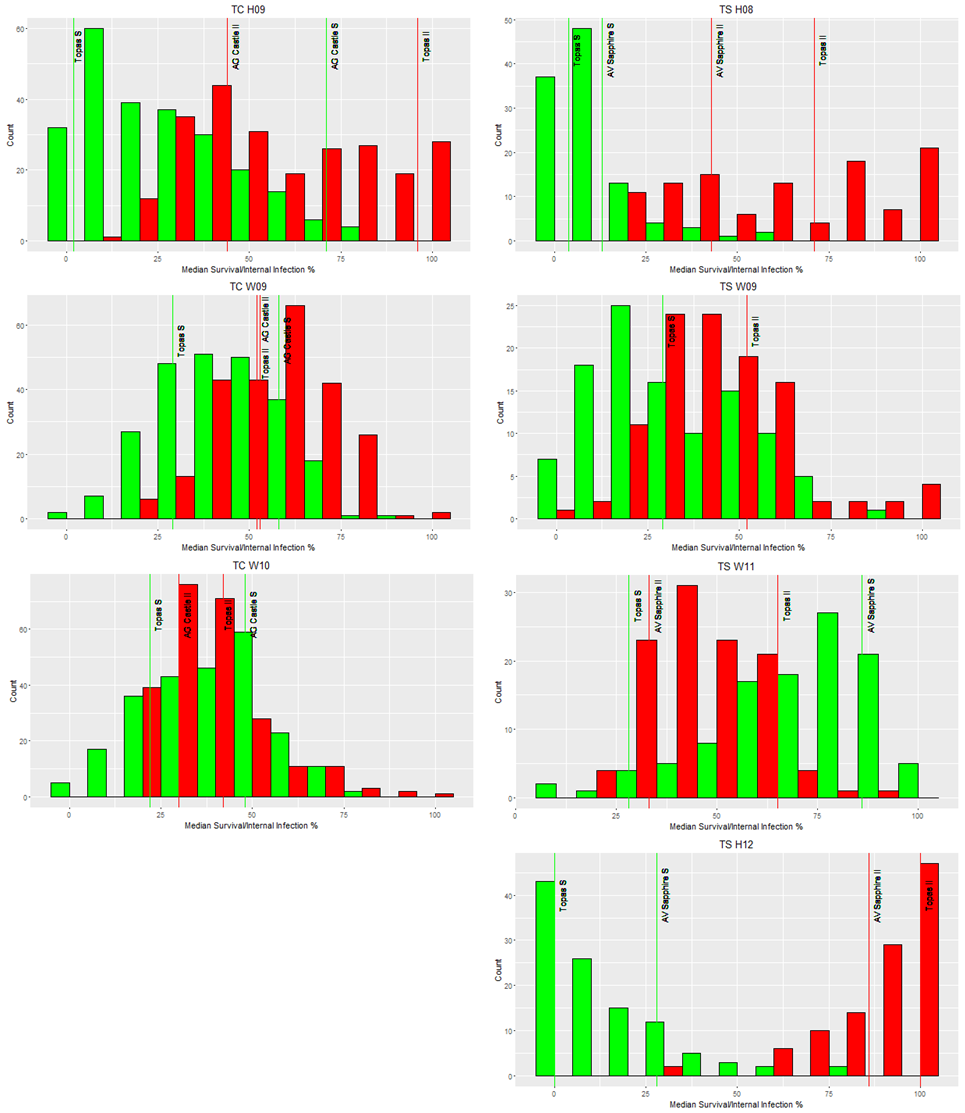

Supplement: Additional file 1: Figure S1. — Distribution of Median Survival and Internal Infection Percentages. Distribution of scores for survival (green bars) and internal infection (red bars) given for each population (TC or TS) in each trial. Environment names given as location (H = Horsham, W = Wagga Wagga), year (08–12 = 2008–2012). Green and red lines indicate mean survival and internal infection percentages for parental lines, respectively. (PNG 127 kb) [file 12870_2016_877_MOESM1_ESM.png]

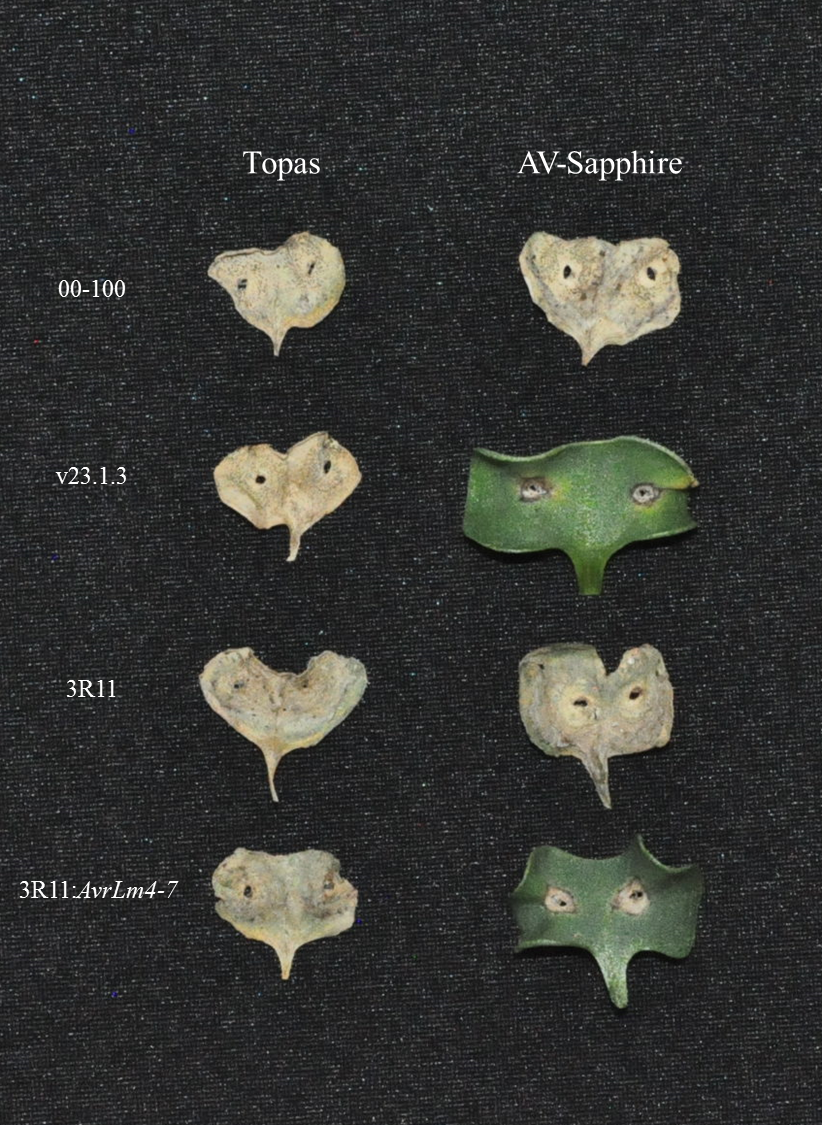

Supplement: Additional file 3: Figure S2. — Transgenic complementation of Rlm4 in AV-Sapphire. Phenotypic interaction of isolates 00–100 (avrLm4–7), v23.1.3 (AvrLm4–7), 3R11 (AvrLm7) and transgenic isolate 3R11: AvrLm4–7 (AvrLm4–7) with B. napus lines Topas (no blackleg R genes) and AV-Sapphire (Rlm4). (PNG 1656 kb) [file 12870_2016_877_MOESM3_ESM.png]
