# Supplementary material for: Multi-environment QTL studies suggest a role for cysteine-rich protein kinase genes in quantitative resistance to blackleg disease in Brassica napus
Source: BMC Plant Biol. 2016 Aug 24;16(1):183. doi: 10.1186/s12870-016-0877-2 (PMC4995785; doi:10.1186/s12870-016-0877-2)
Supplement: Additional file 2: Table S1. — All single- and multi-environment QTL (LOD >2.5). (DOCX 43 kb) [file 12870_2016_877_MOESM2_ESM.docx]

Supplementary Table 1. All single- and multi-environment QTL (LOD >2.5)

A) TC Population

| **Trait** | **Chrom.** | **Peak cM** | **Peak Interval** | **LOD** | **σ²(%)** | **Add** | **σ²_A_** | **σ²_E_** | ***h*^2^** |
| --- | --- | --- | --- | --- | --- | --- | --- | --- | --- |
|  |  |  |  |  |  |  |  |  |  |
| MET S | A01 | 58.5 | sR9564-sN11665 | 6.38 | 2.64 | 2.41 | 1.88 | 0.76 | 0.71 |
| W09 S | A01 | 59 | sN11665-sN12790 | 5.20 | 8.32 | 4.61 |  |  |  |
| H09 S | A01 | 69 | sR8420-sN4638 | 17.20 | 15.84 | 7.80 |  |  |  |
| W10 S | A01 | 69 | sR8420-sN4638 | 24.25 | 29.90 | 9.31 |  |  |  |
| W10 II | A01 | 69 | sR8420-sN4638 | 9.08 | 11.52 | 4.90 |  |  |  |
| MET II | A01 | 69 | sR8420-sN4638 | 34.85 | 20.55 | 6.53 | 12.06 | 8.49 | 0.59 |
| MET S | A01 | 69 | sR8420-sN4638 | 41.47 | 15.84 | 5.79 | 10.80 | 5.04 | 0.68 |
| H09 II | A01 | 69.5 | sN4638-sN12176 | 25.71 | 31.77 | 14.01 |  |  |  |
| H09 S | A01 | 111.5 | brPb-662007-sN3523R | 7.34 | 6.17 | 4.87 |  |  |  |
| MET S | A01 | 111.5 | brPb-662007-sN3523R | 7.35 | 2.53 | 1.56 | 0.78 | 1.75 | 0.31 |
|  |  |  |  |  |  |  |  |  |  |
| CotQTL | A07 | 73.5 | *Rlm3-sR12829* | 72.89 | 55.71 | 20.46 |  |  |  |
|  |  |  |  |  |  |  |  |  |  |
| W10 II | A08 | 15.5 | sN12264a-sNRF19a | 2.84 | 3.40 | 2.67 |  |  |  |
| H09 S | A08 | 39 | sN4513Fa-sNRG04 | 8.96 | 7.63 | 5.49 |  |  |  |
| W09 S | A08 | 39 | sN4513Fa-sNRG04 | 4.45 | 7.05 | 4.32 |  |  |  |
| MET S | A08 | 39 | sN4513Fa-sNRG04 | 13.59 | 5.13 | 3.51 | 3.85 | 1.28 | 0.75 |
| H09 II | A08 | 41 | sNRG04-sNRB88 | 7.42 | 7.85 | 7.08 |  |  |  |
| MET II | A08 | 41.5 | sNRG04-sNRB88 | 10.29 | 5.26 | 3.39 | 3.16 | 2.10 | 0.60 |
| W09 II | A08 | 43 | sNRB88-sN12352a | 3.00 | 4.97 | 3.48 |  |  |  |
| W10 S | A08 | 46 | sNRB88-sN12352a | 8.34 | 9.08 | 5.19 |  |  |  |
| W10 II | A08 | 54.5 | sR11332-sN0202a | 4.07 | 4.97 | 3.22 |  |  |  |
| MET II | A08 | 54.5 | sR11332-sN0202a | 4.13 | 0.99 | 1.32 | 0.49 | 0.49 | 0.50 |
|  |  |  |  |  |  |  |  |  |  |
| W09 II | A10 | 114 | sORH62-sR12228a | 2.66 | 4.44 | 3.24 |  |  |  |
|  |  |  |  |  |  |  |  |  |  |
| W10 II | C03 | 61 | sR12057b-sN11587a | 3.84 | 4.66 | -3.14 |  |  |  |
| MET II | C03 | 61 | sR12057b-sN11587a | 6.40 | 2.18 | -2.69 | 2.01 | 0.17 | 0.92 |
| MET S | C03 | 65 | sR12057b-sN11587a | 6.33 | 2.16 | -2.53 | 2.02 | 0.14 | 0.93 |
| H09 S | C03 | 71 | sR12057b-sN11587a | 4.31 | 4.40 | -4.17 |  |  |  |
|  |  |  |  |  |  |  |  |  |  |
| MET S | C04 | 36.5 | brPb-663380-sN11672a | 4.79 | 1.58 | -2.12 | 1.42 | 0.16 | 0.90 |
| H09 S | C04 | 37 | brPb-663380-sN11672a | 2.95 | 2.70 | -3.26 |  |  |  |
| MET II | C04 | 43 | sN11672a-sR12610b | 7.69 | 2.74 | -3.04 | 2.53 | 0.20 | 0.93 |
| W10 II | C04 | 44.5 | sN11672a-sR12610b | 4.40 | 5.74 | -3.50 |  |  |  |
|  |  |  |  |  |  |  |  |  |  |
| H09 II | C05 | 91.5 | sN12804-brPb-660192 | 3.74 | 3.69 | 4.77 |  |  |  |
| MET II | C05 | 91.5 | sN12804-brPb-660192 | 8.86 | 3.42 | 3.19 | 2.89 | 0.54 | 0.84 |
| W10 II | C05 | 93.5 | sN12804-brPb-660192 | 4.65 | 6.31 | 3.62 |  |  |  |
| W09 S | C05 | 129.5 | sORE94x-sR9555b | 2.75 | 4.27 | 3.30 |  |  |  |
|  |  |  |  |  |  |  |  |  |  |
| W09 II | C06b | 21.5 | brPb-841625-brPb-841355 | 3.04 | 5.17 | 3.50 |  |  |  |
| H09 S | C06b | 40 | brPb-841355-sN12461Ix | 14.59 | 14.08 | 7.42 |  |  |  |
| CotQTL | C06b | 40 | brPb-841355-sN12461Ix | 4.71 | 9.26 | 7.25 |  |  |  |
| MET S | C06b | 42.5 | brPb-841355-sN12461Ix | 25.99 | 9.22 | 5.06 | 8.12 | 1.10 | 0.88 |
| W10 II | C06b | 44.5 | brPb-841355-sN12461Ix | 5.88 | 7.35 | 3.94 |  |  |  |
| H09 II | C06b | 45 | brPb-841355-sN12461Ix | 10.44 | 11.07 | 8.34 |  |  |  |
| W10 S | C06b | 45 | brPb-841355-sN12461Ix | 10.00 | 10.83 | 5.65 |  |  |  |
| MET II | C06b | 45 | brPb-841355-sN12461Ix | 16.44 | 7.83 | 4.30 | 5.14 | 2.70 | 0.66 |
|  |  |  |  |  |  |  |  |  |  |
| CotQTL | C07 | 81 | sS2485-sN12508Ia | 2.65 | 1.38 | 2.77 |  |  |  |
| H09 S | C07 | 103 | sN11863a-sR12127a | 3.09 | 2.48 | 3.08 |  |  |  |

B) TS Population

| **Trait** | **Chrom.** | **Peak cM** | **Peak Interval** | **LOD** | **σ²(%)** | **Add** | **σ²_A_** | **σ²_E_** | ***h*^2^** |
| --- | --- | --- | --- | --- | --- | --- | --- | --- | --- |
|  |  |  |  |  |  |  |  |  |  |
| W09 S | A01 | 24 | sR9481a-sR1377b | 2.58 | 11.60 | 6.69 |  |  |  |
| H12 S | A01 | 36.5 | sR6202b-sR9555x | 4.39 | 10.10 | 5.33 |  |  |  |
| MET S | A01 | 36.5 | sR6202b-sR9555x | 13.17 | 10.07 | 5.04 | 8.71 | 1.36 | 0.86 |
| W11 II | A01 | 37.5 | sR6202b-sR9555x | 3.44 | 11.07 | 4.39 |  |  |  |
| W11 S | A01 | 39 | sR6202b-sR9555x | 6.35 | 18.47 | 8.21 |  |  |  |
| H12 II | A01 | 39.5 | sR9555x-sN12176 | 3.37 | 9.00 | 4.08 |  |  |  |
| MET II | A01 | 40 | sR9555x-sN12176 | 7.83 | 5.02 | 4.20 | 4.74 | 0.28 | 0.94 |
|  |  |  |  |  |  |  |  |  |  |
| MET II | A02 | 14 | sS2372a-sN3761b | 3.19 | 2.81 | 0.21 | 0.01 | 2.80 | 0.00 |
|  |  |  |  |  |  |  |  |  |  |
| MET S | A03 | 22.5 | sR12015a-sN12574b | 6.18 | 4.73 | -3.29 | 3.69 | 1.04 | 0.78 |
| W11 S | A03 | 23.5 | sR12015a-sN12574b | 3.96 | 11.14 | -6.37 |  |  |  |
| W11 II | A03 | 66 | sN6689b-sORE01b | 4.44 | 14.04 | -4.93 |  |  |  |
| MET II | A03 | 66 | sN6689b-sORE01b | 7.01 | 4.23 | -3.50 | 3.31 | 0.92 | 0.78 |
|  |  |  |  |  |  |  |  |  |  |
| H12 II | A03b | 0 | sR12137Ib-sN4034a | 3.61 | 9.72 | -4.25 |  |  |  |
| H12 S | A03b | 0 | sR12137Ib-sN4034a | 3.32 | 8.45 | -4.90 |  |  |  |
| MET S | A03b | 0 | sR12137Ib-sN4034a | 5.65 | 3.86 | -2.87 | 2.80 | 1.06 | 0.72 |
| MET II | A03b | 0 | sR12137Ib-sN4034a | 5.18 | 2.52 | -2.86 | 2.17 | 0.35 | 0.86 |
|  |  |  |  |  |  |  |  |  |  |
| CotQTL | A07 | 29.5 | sN0658-sN2433a | 4.15 | 3.75 | 5.33 |  |  |  |
| CotQTL | A07 | 56 | sN2555Ra-*Rlm4* | 30.80 | 48.92 | 19.16 |  |  |  |
| CotQTL | A07 | 72 | sN9539a-sR0293a | 3.87 | 3.40 | 5.26 |  |  |  |
|  |  |  |  |  |  |  |  |  |  |
| W11 S | A08 | 40 | sN12352a-sORD13x | 2.69 | 7.11 | 5.30 |  |  |  |
| MET S | A08 | 40 | sN12352a-sORD13x | 3.32 | 2.84 | 0.91 | 0.26 | 2.58 | 0.09 |
|  |  |  |  |  |  |  |  |  |  |
| W11 II | A09 | 8 | sR6410-sR9373 | 5.35 | 16.38 | 5.41 |  |  |  |
| H12 II | A09 | 8 | sR6410-sR9373 | 3.05 | 8.12 | 3.92 |  |  |  |
| W09 S | A09 | 8 | sR6410-sR9373 | 2.83 | 11.50 | 6.79 |  |  |  |
| W11 S | A09 | 8 | sR6410-sR9373 | 5.34 | 15.00 | 7.48 |  |  |  |
| MET S | A09 | 8 | sR6410-sR9373 | 10.41 | 9.18 | 4.67 | 7.33 | 1.86 | 0.80 |
| W09 II | A09 | 8.5 | sR6410-sR9373 | 2.89 | 11.83 | 6.68 |  |  |  |
| MET II | A09 | 9.5 | sR6410-sR9373 | 13.97 | 9.66 | 5.88 | 9.10 | 0.56 | 0.94 |
| H08 II | A09 | 25 | sR9373-sR6966 | 3.43 | 12.31 | 9.82 |  |  |  |
| H12 S | A09 | 27.5 | sR9373-sR6966 | 3.41 | 9.47 | 5.19 |  |  |  |
| MET S | A09 | 29 | sR9373-sR6966 | 6.05 | 3.32 | 2.63 | 2.36 | 0.96 | 0.71 |
|  |  |  |  |  |  |  |  |  |  |
| MET S | C01 | 13 | sN11641b-sR12341a | 4.40 | 3.59 | -2.95 | 2.99 | 0.61 | 0.83 |
| MET S | C01 | 33.5 | sR6762a-sNRA84 | 6.42 | 3.51 | -3.13 | 3.32 | 0.19 | 0.95 |
| MET II | C01 | 34 | sNRA84-sR9228b | 4.53 | 3.72 | -3.40 | 3.09 | 0.63 | 0.83 |
| H08 S | C01 | 34.5 | sNRA84-sR9228b | 3.90 | 12.90 | -4.39 |  |  |  |
| MET II | C01 | 53 | sR6993Fa-sN9425 | 4.10 | 2.96 | -2.95 | 2.36 | 0.60 | 0.80 |
| MET S | C01 | 90.5 | sN3749a-sR0686b | 3.08 | 1.54 | -1.59 | 0.86 | 0.68 | 0.56 |
| MET II | C01 | 91 | sN3749a-sR0686b | 4.81 | 3.45 | -3.07 | 2.51 | 0.94 | 0.73 |
|  |  |  |  |  |  |  |  |  |  |
| MET II | C04 | 100 | sORD34-sN8093Ia | 3.42 | 3.58 | 2.91 | 2.27 | 1.31 | 0.63 |
|  |  |  |  |  |  |  |  |  |  |
| H08 II | C06 | 52.5 | sS2486a-sR12387a | 4.79 | 19.31 | 12.24 |  |  |  |
| H12 S | C06 | 54.5 | sS2486a-sR12387a | 6.91 | 22.18 | 7.88 |  |  |  |
| H12 II | C06 | 56.5 | sS2486a-sR12387a | 4.55 | 13.63 | 5.00 |  |  |  |
| MET II | C06 | 57 | sS2486a-sR12387a | 10.84 | 9.67 | 4.97 | 6.69 | 2.98 | 0.69 |
| H08 S | C06 | 58 | sS2486a-sR12387a | 4.58 | 15.26 | 4.73 |  |  |  |
| MET S | C06 | 58 | sS2486a-sR12387a | 14.65 | 9.34 | 5.04 | 8.76 | 0.58 | 0.94 |

C) LOD significance thresholds determined by permutation tests

| TC | CotQTL | 3.0345 |
| --- | --- | --- |
|  | H09II | 3.0717 |
|  | H09S | 2.9272 |
|  | W09II | 3.0099 |
|  | W09S | 2.7558 |
|  | W10II | 2.8686 |
|  | W10S | 2.9593 |
|  |  |  |
| TS | CotQTL | 2.9992 |
|  | H08II | 2.9507 |
|  | H08S | 2.5453 |
|  | W09II | 2.8154 |
|  | W09S | 2.9286 |
|  | W11II | 2.9103 |
|  | W11S | 2.8802 |
|  | H12II | 2.6639 |
|  | H12S | 2.7328 |

All QTL detected from each environment with LOD > 2.5 for A) Topas/AG-Castle population and B) Topas/AV-Sapphire population. Single environment and multi-environment QTL shown, single-environment trait names given as location (H = Horsham, W = Wagga Wagga), year (08-12 = 2008 – 2012) and metric (S = survival, II = internal infection), MET = Multi-environment traits (all environments) for S (survival) and MET II (internal infection) metrics, CotQTL = single-isolate cotyledon tests. Chrom. = *B. napus* chromosome; Peak (cM) = Position of Peak LOD value (in centiMorgans); Peak Interval = map interval containing QTL peak LOD; LOD = peak logarithm of odds; σ²(%) = variance (total percentage); Add = additive effect (positive score indicates net genetic contribution from AG-Castle or AV-Sapphire parent); σ²_A_ = variance (additive) portion (%), σ²_E_ = variance (environmental) portion (%); *h*^2^ = heritability (σ²_A_/ σ² (%)). Names in red are QTL that failed to meet all significance criteria, values in red are < significance threshold. C) LOD significance thresholds determined for each dataset by permutation test (1000 permutations).
