# Supplementary material for: Multi-environment QTL studies suggest a role for cysteine-rich protein kinase genes in quantitative resistance to blackleg disease in Brassica napus
Source: BMC Plant Biol. 2016 Aug 24;16(1):183. doi: 10.1186/s12870-016-0877-2 (PMC4995785; doi:10.1186/s12870-016-0877-2)
Supplement: Additional file 4: Table S2. — Survival and internal infection of control B. napus lines in each environment. (DOCX 16 kb) [file 12870_2016_877_MOESM4_ESM.docx]

Supplementary Table 2. Survival and internal infection of control *B. napus* lines in each environment

| a) Survival |  | H08 | W09 | H09 | W10 | W11 | H12 |
| --- | --- | --- | --- | --- | --- | --- | --- |
|  |  |  |  |  |  |  |  |
| Topas |  | 4.4% | 28.8% | 1.9% | 21.8% | 28.3% | 0.0% |
| AV-Sapphire | | 13.2% | n/a | - | - | 86.0% | 28.1% |
| AG-Castle |  | - | 57.9% | 70.8% | 48.4% | - | - |
|  |  |  |  |  |  |  |  |
| Westar |  | - | 1.7% | - | 0.4% | 14.9% | 0.0% |
| AV-Garnet |  | 65.0% | 42.3% | 23.8% | - | 96.4% | 49.1% |
| Hyola 50 |  | 89.0% | 68.2% | 68.2% | - | 89.7% | 88.3% |
|  |  |  |  |  |  |  |  |
|  |  |  |  |  |  |  |  |
| b) Internal Infection | | H08 | W09 | H09 | W10 | W11 | H12 |
|  |  |  |  |  |  |  |  |
| Topas |  | 71.0% | 52.0% | 96.0% | 42.0% | 65.0% | 100.0% |
| AV-Sapphire | | 43.0% | n/a | - | - | 33.0% | 86.0% |
| AG-Castle |  | - | 52.0% | 43.6% | 30.0% | - | - |
|  |  |  |  |  |  |  |  |
| Westar |  | - | 95.0% | - | 98.0% | 77.0% | 100.0% |
| AV-Garnet |  | 24.0% | 26.0% | 32.0% | - | 36.0% | 66.0% |
| Hyola 50 |  | 1.0% | 13.0% | 16.0% | - | 15.0% | 14.0% |
|  |  |  |  |  |  |  |  |

Mean a) survival and b) internal infection percentages for *B. napus* controls in each environment. Environment names given as location (H = Horsham, W = Wagga Wagga), year (08-12 = 2008 – 2012)
