# Supplementary material for: Multi-environment QTL studies suggest a role for cysteine-rich protein kinase genes in quantitative resistance to blackleg disease in Brassica napus
Source: BMC Plant Biol. 2016 Aug 24;16(1):183. doi: 10.1186/s12870-016-0877-2 (PMC4995785; doi:10.1186/s12870-016-0877-2)
Supplement: Additional file 5: Table S3. — Rainfall (mm) for trial sites over growing season (May–Nov). (DOCX 15 kb) [file 12870_2016_877_MOESM5_ESM.docx]

Supplementary Table 3. Rainfall (mm) for trial sites over growing season (May – Nov).

| **Location** | **Year** | **May** | **Jun** | **Jul** | **Aug** | **Sep** | **Oct** | **Nov** | **Total - Growing Season** | **3 Season Average** |
| --- | --- | --- | --- | --- | --- | --- | --- | --- | --- | --- |
| **Horsham** | 2008 | 37.8 | 33.8 | 50.8 | 36.4 | 21.4 | 5.6 | 11.6 | 197.4 | 271.5 |
|  | 2009 | 59.8 | 58.2 | 62 | 49.2 | 83 | 21.2 | 61.8 | 395.2 |  |
|  | 2012 | 31.6 | 53 | 48 | 31.8 | 31.6 | 13.6 | 12.4 | 222 |  |
|  |  |  |  |  |  |  |  |  |  |  |
| **Wagga Wagga** | 2009 | 8.2 | 47.4 | 36.2 | 33 | 33.2 | 25.8 | 27.4 | 211.2 | 374.7 |
|  | 2010 | 68.2 | 38 | 74.4 | 96.4 | 65 | 170.2 | 50.6 | 562.8 |  |
|  | 2011 | 31 | 23.4 | 35.2 | 49 | 40.2 | 18.8 | 152.4 | 350 |  |
